# Age-related effects of body mass on fertility and litter size in roe deer

**DOI:** 10.1371/journal.pone.0175579

**Published:** 2017-04-12

**Authors:** Katarina Flajšman, Klemen Jerina, Boštjan Pokorny

**Affiliations:** 1 Slovenian Forestry Institute, Ljubljana, Slovenia; 2 Department of Forestry and Renewable Forest Resources, Biotechnical Faculty, University of Ljubljana, Ljubljana, Slovenia; 3 Environmental Protection College, Velenje, Slovenia; 4 Ecological Research and Industrial Cooperation, Velenje, Slovenia; Université de Sherbrooke, CANADA

## Abstract

We analysed effects of females’ body mass and age on reproductive capacity of European roe deer (*Capreolus capreolus*) in a large sample set of 1312 females (305 yearlings and 1007 adults), hunted throughout Slovenia, central Europe, in the period 2013–2015. Body mass positively affected probability of ovulation and potential litter size (number of corpora lutea), although its effect was more pronounced in yearlings than in adults. Between age groups, we found clear differences in responses of both reproductive parameters to body mass which influences primarily reproductive performance of younger, and in particular, lighter individuals: at the same body mass yearlings would at average have smaller litters than adults, and at lower body mass also young to middle-aged adults would have smaller litters than old ones. In addition, while yearlings have to reach a critical threshold body mass to attain reproductive maturity, adult females are fertile (produce ova) even at low body mass. However, at higher body mass also younger individuals shift their efforts into the reproduction, and after reaching an age-specific threshold the body mass does not have any further effects on the reproductive output of roe deer females. Increased reproductive capacity at more advanced age, combined with declining body mass suggests that old does allocate more of their resources in reproduction than in body condition.

## Introduction

The European roe deer (*Capreolus capreolus*) has one of the largest distribution ranges among wild ungulates and is widespread and abundant almost all over the European continent [1, 2]. Across its pan-European distribution, the species faces a wide diversity of environmental and climatic conditions, so several factors shape its life-history traits and cause high variability in some of the most important parameters of reproductive success, e.g. fertility (the ability to produce ova, i.e. to ovulate) and litter size (reviewed in [3]). Apart from population density (e.g. [4–8]) and different environmental factors [8–11], individual characteristics determining physical condition of does have a very important influence on their reproductive performance (e.g. [4, 12–16]).

Roe deer are the only artiodactyls to display embryonic diapause [17] which enables them to mate and to give birth in the parts of the year with favourable conditions [18]. As a polytocous species, roe deer females face high energy demands after implantation and during pregnancy [19]. During the rut, roe deer does are still suckling the previous year’s fawns, and due to the costs of lactation in spring they are likely to be in poorer body condition at that time [4]. Moreover, roe deer is typical income breeder with few available body reserves [20]. In consequence, body mass as a proxy of individual condition and quality is particularly informative in roe deer and is of crucial importance in determining the reproductive success of the species (see [21]).

Body mass affects a number of different components of roe deer reproductive potential: (i) In the first instance body mass is crucial for females to reach reproductive maturity [6]. To reach puberty and breed for the first time females have to reach a threshold body mass [13] but the first reproduction usually occurs before does have reached their full body size [22]. (ii) In both subadults and adults, heavier females have higher ovulation rate and produce larger litters [6, 7, 19].

The effect of body mass on reproductive potential of roe deer females has been studied in several populations across Europe (e.g. [4, 6, 7, 12, 15]; reviewed in [3]). Apart from some rare studies (e.g. [4]) age-related effects have generally been explored by contrasting two main age groups, yearlings and adult does. In the current study (based on a large sample of roe deer females hunted throughout Slovenia, central Europe) we aimed to find whether there are any differences in responses of fertility and potential litter size to body mass variation between those two age groups. We predicted that body mass would positively affect both reproductive parameters, but with much more pronounced effects in yearlings.

In long-lived mammals, costs of reproduction may vary with age and both body mass and reproductive effort change during the animal’s lifetime. Roe deer females generally produce their first offspring before they reach their full body size [22]. Primiparous does therefore allocate their resources primarily into continued body growth [23]. With ageing, body mass may decline due to the senescence of physiological functions and decreasing foraging ability [21, 24] linked to advancing tooth-wear [25, 26]. However, the reproductive output may either decline due to the same senescence [27] or may actually increase due to selection (assuming that the individuals with higher vitality, i.e. better reproductive performance would survive the longest) or greater reproductive effort as females approaching the end of their life expectancy [28, 29].

Mothers might invest more in reproduction as they age [30], and older females have fewer resources to allocate to reproduction due to physiological decline with ageing [31]. While ovulation rate is an important parameter of reproductive performance (ovulation determines a possibility that a female becomes pregnant, and number of released ova limits the maximal litter size), implantation and maternal care during suckling (determining also juvenile survival) are the most variable parameters in terms of individual investment strategy [32, 33]. It has been reported that in senescent animals of different deer species the reproductive rate/output decreases, i.e. in red deer (*Cervus elaphus*) [34, 35] and fallow deer (*Dama dama*) [36]. In roe deer, also a decline in reproductive rate with age has been reported–whether due to an overall decrease in fertility [37], smaller litters [38] or higher implantation failure in senescent females [19, 39]. To the best of our knowledge, however, these are the only studies that describe the effect of aging on roe deer reproduction. Therefore, apart from investigating the effect of body mass on reproductive performance of roe deer females in two main age groups (yearlings and adults) we also explored changes in reproductive potential in different age classes of adult does. Since sampling was made within regular hunting operations, i.e. during the autumn when roe deer females are in embryonic diapause [17], this study is based on counts of corpora lutea in the early stage of pregnancy (before implantation of fertilised ova). Therefore, we neither aimed to determine age-related changes in reproductive investment (ovulation and investment before implantation are of relatively low cost [32, 33]) nor in actual litter size (which may be affected by implantation failure, abortions or neonatal mortality); rather, we tried to determine possible influence of aging on does’ ovulation rate and ability to produce different number of ova(s).

## Methods

All individuals of roe deer used in the study were hunted during the regular hunting activity prescribed by the state of Slovenia within the yearly hunting management plans. We used only tissues of already dead individuals therefore no animal was shot or killed by any other means for the purposes of the research.

### Study area

Reproductive organs of roe deer females were collected in 76 hunting grounds, distributed in 14 hunting management districts continuously throughout Slovenia, covering the whole range of environmental conditions and population characteristics of the species’ distribution in the country. Slovenia (20,273 km^2^) is located in Central Europe (46°N, 14°E) at the junction of four large European geographical units, i.e. the Alps, the Pannonian Basin, the Dinaric Alps, and the Mediterranean region. The climate is diverse, but roughly with a continental climate in the northeast, a severe alpine climate in the high mountain regions, and sub-Mediterranean climate in the coastal region.

The roe deer is the most abundant and the most important game species in Slovenia. Population size is estimated at >200,000 individuals [40]. In the period 2006–2015, annual total recorded mortality (hunted animals, road-kill, diseases, etc.) was between 39,599 and 41,768 animals which sums to 409,650 animals in the 10-year period [41].

### Data collection

Reproductive organs (uteri with ovaries) of female roe deer were sampled in the period 2013–2015, within regular hunting operations (the hunting season for female roe deer runs from 1^st^ September to 31^st^ December). In total, 1896 samples were collected, and 1312 of them were suitable for analysis considering the scope of this paper (i.e. samples with both ovaries, available data on body mass, access to the mandible for age assessment). Immediately after the cull and dissection, hunters placed uteri into plastic bags and stored them frozen until collection; the lower jaw was also removed and the left half of the mandible retained for age assessment. For each specimen, sampling date, location, eviscerated carcass mass (total body mass less viscera but with head and feet on–the eviscerated body masses were used in all analyses and are presented throughout this paper) and age group (yearling, adult) were recorded immediately after the hunting episode. Afterwards, samples were defrosted and analysed in the laboratory of the institute ERICo Velenje and at the Slovenian Forestry Institute. To determine the fertility and potential litter size of each female, the presence and number of corpora lutea (CL) were determined by dissection of ovaries. Fertile females were those that had CL in their ovaries, meaning that they were able to reproduce as they ovulated. The number of CL per female was considered as a potential litter size of each individual (see [42, 43]).

For all animals, age was assessed jointly by the first and the last author of the manuscript by macroscopic inspection of tooth development and tooth-wear in mandible samples accompanying each specimen [43, 44]. Due to known uncertainty in the age assessment of adult roe deer on the basis of tooth wear criteria [45] the age of adults was not determined with a yearly precision. Rather, animals were grouped into six age classes. As criterion for classification, pre-prepared set of mandibles with different and easily distinguished tooth-wear patterns was used. Age class of each individual was defined in consensus of both evaluators, and their separate pre-assessments had differed in <5% of all mandibles (in all cases for one age class only, no discrepancies occurred when distinguishing yearlings from adults). The following age classes were used: yearlings (15–19 months old; n = 305), 2-year-olds (n = 186), young adults (3–4 years; n = 364), middle-aged adults (5–7 years; n = 259), old adults (8–9 years; n = 158), and elderly adults (10+ years; n = 40), respectively.

### Data analysis

Considering the origin of the samples, all analyses were done on cross-sectional data of different animals measured only once, after they were hunted. Before performing analyses on reproductive parameters, we checked whether the body mass of roe deer females differed through the sampling period from 1^st^ September to 31^st^ December. As body mass of both yearlings and adults linearly increased with consecutive day in the year, we performed correction of this variable using a General Regression Model (see S1 Text and S1 Table). All further analyses were done with corrected eviscerated body masses.

To explore the reproductive potential of roe deer females at the population level, as in several previous studies (for review, see [3]), we used various parameters/measures: (i) fertility (fertile and non-fertile individuals; later: *fertility*), (ii) mean number of CL in fertile individuals (later: *potential litter size*), and (iii) synthesis of both of the above parameters–mean number of CL, including also non-fertile individuals (later: *potential reproductive output*). All statistical analyses were performed using the first two parameters (fertility and potential litter size), while the third parameter (potential reproductive output) was used in all graphical presentations.

**Fig 1:**
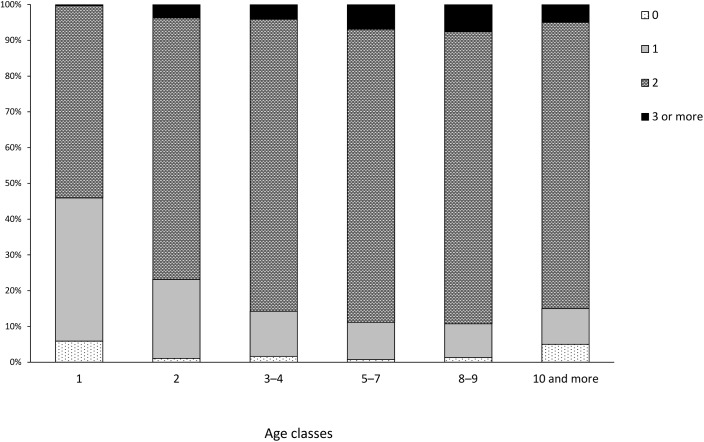
Relative frequencies of the number of CL in roe deer females of different age classes in Slovenia in the period 2013–2015 (n = 1312). Note that age was estimated using macroscopic inspection of tooth-wear, a method that is less accurate (especially) in older animals.

**Fig 2:**
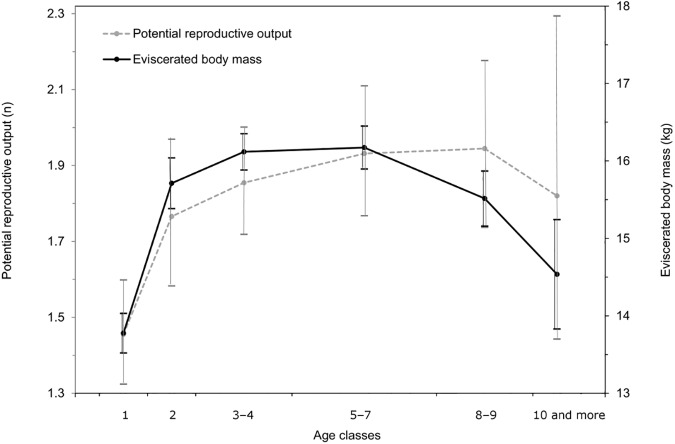
Age-dependent differences in mean eviscerated body mass and potential reproductive output (number of CL, including infertile individuals) in roe deer females in Slovenia (n = 1312). Error bars denote 95% confidence intervals of the mean. Note that age was estimated using macroscopic inspection of tooth-wear, a method that is less accurate (especially) in older animals.

To visualize differences in potential reproductive output among females of different ages, we calculated the relative frequencies of the number of CL per doe (0, 1, 2, 3, 4, 5) for each of the age classes and plotted them in Fig 1. We also calculated and visualised the mean with confidence intervals (p = 0.05) for potential reproductive output and body mass for all age classes (Fig 2). When calculating the confidence intervals, we assumed the normal distribution of body mass and Poisson distribution of the number of CL. Data on the frequency of the number of CL in different body mass classes are presented separately for yearlings and adults in S2 Table.

Prior to statistical analyses, we grouped all individuals into three age categories in order to minimize possible errors in the age assessment, taking into account age-specific changes in both body mass and potential reproductive output (Fig 2): yearlings (n = 305), young to middle-aged adults (2–7 years; n = 809) and older adults (8+ years; n = 198). These age categories were used in all statistical analyses.

The effects of age and body mass on reproductive potential were firstly analysed by the chi-square test for homogeneity (Table 1). We analysed fertility and potential litter size separately, as these parameters may respond differently to impact factors (e.g. [19]), and explored differences between yearlings and adults as well as between age groups of adults. Therefore, we performed four separate tests for homogeneity covering all combinations of the studied factors as follows: (i) yearlings *vs*. adults × fertile *vs*. non-fertile; (ii) age class of adults × fertile *vs*. non-fertile; (iii) yearlings *vs*. adults × potential litter size; (iv) age class of adults × potential litter size.

**Table 1:** Bivariate analyses of the effects of age and body mass on the reproductive potential (fertility, potential litter size) of roe deer females in Slovenia. Relations between variables (their categories are listed in parentheses) were analysed by tests for homogeneity. The results of each of eight analyses are presented in each row.

Variable 1	Variable 2	Pearson chi-square value	N	df	*P*	Note
*Age* (yearlings, adults)	*Fertility* (yes, no)	18.2*	1312	1	<<0.001	All individuals
*Age* (2–7, 8+)	*Fertility* (yes, no)	0.7*	1007	1	0.398 (ns)	Only adults
*Age* (yearlings, adults)	*Litter size* (1, 2, 3, 4+)	125.1	1280	3	<<0.001	Only fertile individuals
*Age (2–7*, *8+)*	*Litter size* (1, 2, 3, 4+)	6.3	993	3	0.097 (ns)	Only fertile adults
*Body mass–kg* (<12, 12–14, 14–16, 16–18, 18+)	*Fertility* (yes, no)	111.0	1312	4	<<0.001	All individuals
*Body mass–kg* (<14, 14–16, 16–18, 18+)	*Fertility* (yes, no)	15.5	1007	3	0.001	Only adults
*Body mass–kg* (<12, 12–14, 14–16, 16–18, 18+)	*Litter size* (1, 2, 3+)	182.0	1280	8	<<0.001	Only fertile individuals
*Body mass–kg* (<12, 12–14, 14–16, 16–18, 18+)	*Litter size* (1, 2, 3+)	73.9	993	8	<<0.001	Only fertile adults

* With correction for continuity.

A similar procedure was used to analyse the effects of body mass on reproductive potential. For this analysis, we grouped all individuals into five body mass classes as follows: <12 kg (n = 106); 12–13.9 kg (n = 263); 14–15.9 kg (n = 395); 16–17.9 kg (n = 351); and >18 kg (n = 197). We analysed the following combinations of variables: (v) body mass × fertility for all individuals; (vi) body mass × fertility for adults; (vii) body mass × potential litter size for all individuals; (viii) body mass × potential litter size for adults. To meet the criteria of minimal theoretical frequency, required by the chi-square test for homogeneity, we pooled the first two body mass classes into one (<14 kg; n = 369) prior to analysis (vi), and pooled all females with the number of CL = 3 (n = 48), 4 (n = 5) and 5 (n = 1) into one group (CL = 3 or more) prior to analyses (vii) and (viii).

**Fig 3:**
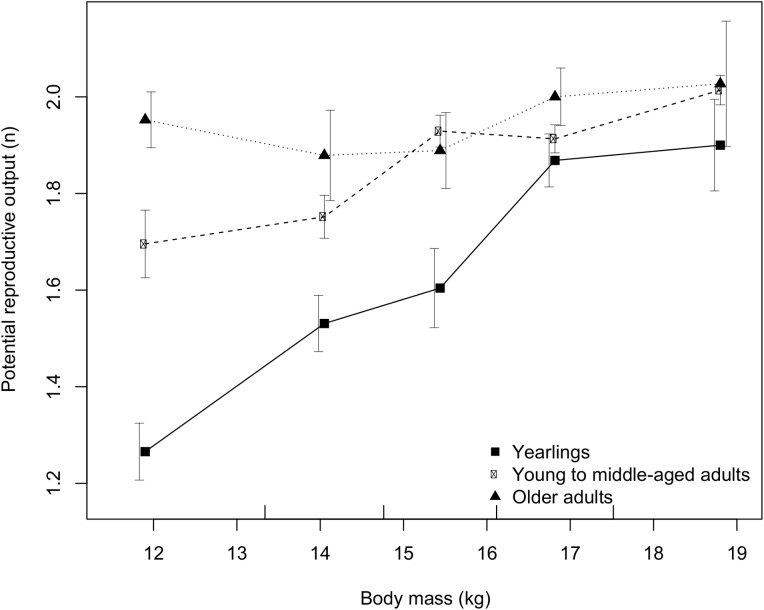
Potential reproductive output (number of CL, including infertile individuals) of roe deer females in relation to body mass by age category. Age categories are as follows: yearlings (15–19 months old), young to middle-aged adults (2–7 years old), and older adults (8+ years old). Samples were pooled based on the body mass of individuals into five groups with an equal number of units across the total sample set (for intervals, see marks above the X axis). In the case of the first and the last body mass groups, dots are horizontally positioned on the mean body mass of all individuals in these two groups. Error bars denote 95% confidence intervals of the mean.

The effects of body mass on reproductive potential could be age specific (i.e. the interaction between variables has to be explored), but homogeneity tests are not convenient to explore these interactions/effects. Therefore, we also analysed our data with generalized linear models (GLM). As in the homogeneity tests, we used fertility (fertile *vs*. non-fertile; binomial error) and potential litter size (number of CL = 1–5; Poisson distribution of the error) as dependent variables and explored differences in the effects of both yearlings *vs*. adults, and between both age categories of adults. In all GLM analyses in addition to age (fixed factor), the independent variables were body mass (covariate) and the interaction of body mass × age; we also explored the effects of the year of sampling (2013, 2014, 2015) as a fixed factor. We built all possible models and used the Akaike information criteria (AIC) to select the best and other still informative models with ΔAIC < 2. We displayed parameter estimates and other base statistics only for the best model, while for the other models we showed only the model structure and ΔAIC (Table 2; Table 3). We also visualised the effects of body mass on potential reproductive output for each of three age categories (Fig 3).

**Table 2:** Generalized linear models of fertility (n = 1312) and potential litter size (n = 1280) of subadult and adult roe deer females in Slovenia (2013–2015). The independent variables were age (yearlings *vs*. adults), body mass (covariate), body mass × age interaction, and year (2013, 2014 *vs*. 2015; fixed factor). Model selection was performed by the Akaike information criteria (AIC). For the best model, basic statistics are displayed, while for other models with ΔAIC < 2 only the model structure and ΔAIC are shown.

**Fertility (best model)** = f (age + body mass + age × body mass + year); AIC = 246.2; **p << 0.001**					
	Estimate	Standard error	Wald value		P value
Age (yearlings *vs*. adults)	-4.727	1.244	14.4		<<0.001
Body mass	0.416	0.094	19.6		<<0.001
Body mass × age (yearlings *vs*. adults)	0.332	0.094	12.5		<<0.001
Year (2013 *vs*. 2015)	-0.781	0.285	7.5		0.006
Year (2014 *vs*. 2015)	-0.409	0.281	2.1		0.146
Other models with ΔAIC < 2: none					
**Potential litter size (best model)** = f (age + body mass + age × body mass); AIC = 2023.3; **p << 0.001**					
	Estimate	Standard error		Wald value	P value
Age (yearlings *vs*. adults)	-0.279	0.178		2.5	0.117
Body mass	0.035	0.012		8.5	0.004
Body mass × age (yearlings *vs*. adults)	0.014	0.012		1.4	0.243
Other models with ΔAIC < 2: f (age + body mass), ΔAIC = 0.5; f (age + body mass + year), ΔAIC = 1.9					

**Table 3:** Generalized linear models of fertility (n = 1007) and potential litter size (n = 993) of adult roe deer females in Slovenia (2013–2015). The independent variables were age (2–7 years *vs*. 8+ years), body mass (covariate), body mass × age interaction, and year (2013, 2014 *vs*. 2015; fixed factor). Model selection was performed using the Akaike information criteria (AIC). For the best model, basic statistics are displayed, while for other models with ΔAIC < 2 only the model structure and ΔAIC are shown.

**Fertility (best model)** = f (year); AIC = 142.8; **p = 0.004**				
	Estimate	Standard error	Wald value	P value
Year (2013 *vs*. 2015)	-0.004	0.588	0.001	0.995
Year (2014 *vs*. 2015)	-1.218	0.457	7.098	0.008
Other models with ΔAIC < 2: none				
**Potential litter size (best model)** = f (age + body mass + age × body mass); AIC = 2646.2; **p = 0.012**				
	Estimate	Standard error	Wald value	P value
Age (2–7 years *vs*. 8+ years)	-0.095	0.089	1.4	0.262
Body mass	0.020	0.012	2.6	0.089
Body mass × age (2–7 years *vs*. 8+ years)	0.010	0.016	0.3	0.532
Other models with ΔAIC < 2: f (age + body mass), ΔAIC = 1.1; f (body mass), ΔAIC = 1.9				

We performed all statistical analyses using the lme4 package in R [46] and STATISTICA data analysis software system [47]. To create the graphs, we used Microsoft Excel.

## Results

In the period 2013–2015, the fertility of roe deer females in Slovenia was generally very high (97.0%, regardless of the age of the animals), averaging 94.1% in yearlings, 98.7% in young to middle-aged adults, and 98.0% in older (8+) females, respectively. Among 1312 analysed females, only 30 individuals (2.3%), i.e. 18 yearlings (5.9%) and 12 adults (1.4%), did not ovulate in the year of sampling. A significant increase in fertility was found between yearlings and adults (Table 1; Table 2), while no differences were estimated between age categories of adults either with the homogeneity test (Table 1) or GLM (Table 3).

Fertile females carried 1 (19.9%), 2 (75.8%), 3 (3.8%), 4 (five individuals, 0.4%) or 5 CL (one individual, 0.08%) (Fig 1). The potential litter size generally increased with age. In yearlings (n = 305), only one animal had three CL, but 40% carried only one CL (potential litter size = 1.58 ± 0.06, n = 287). In contrast, almost 7% of middle-aged and 8% of old females carried 3 or more CL (Fig 1). Average potential litter size for adult females was 1.93 ± 0.03 (n = 993); trend suggests a peak in old does (1.97 ± 0.32, n = 158) and slight decrease thereafter (see also Fig 2) but differences between categories of adults were not statistically significant (Table 3); however, 15% of elderly does had only one CL or even failed to ovulate (Fig 1). Similarly as in the case of fertility also potential litter size differed significantly between yearlings and adults (Table 1; Table 2) but not between two age categories of adults (Table 1; Table 3). However, both the age and interaction age × body mass were included in the best GLM of potential litter size for adults (Table 3), indicating that also the age in synergy with of body mass, contributed to variability in the potential litter size/output of adult does (see also Fig 2; Fig 3).

Body mass affected fertility and potential litter size. With increasing body mass, there is a clear increase in reproductive potential of roe deer females, which was confirmed both by the test for homogeneity (Table 1) and the GLM (Table 2). In yearlings, body mass strongly affected both reproductive parameters, but in adults this effect was much weaker. GLM analyses indicated that in adult does body mass affects potential litter size (note also the structure of the GLMs of potential litter size with ΔAIC <2) but not fertility (Table 3). In contrast, the test for homogeneity revealed differences also in fertility among body mass classes in adults (Table 1), but these differences did not follow the body mass gradient and are difficult to interpret.

The mean eviscerated body mass of roe deer females depended on the age of the individual; it increased from 13.8 ± 0.3 kg in yearlings to 16.2 ± 0.3 kg in middle-aged females, where it reached its peak. After this age it started to decrease, and females from the oldest age class (elderly does) on average weighed 14.5 ± 0.7 kg. The largest increase in body mass was observed between yearlings and 2-year-old does (+1.9 kg, 13.8%), confirming that yearlings had still not reached their full body size. While the decrease in body mass was observed in the last two age classes, in the case of reproduction females tend to reach the peak at an older age (Fig 2).

Body mass and age had a synergistic effect on the reproductive parameters of roe deer. In yearlings, 50% of animals with eviscerated body mass <10 kg were infertile, and some infertile individuals were also in body mass categories up to 15.9 kg. In contrast, all (6) yearlings with eviscerated body mass >18.0 kg had two CL. There were some very rare individuals which failed to ovulate among adults, primarily in the body mass category up to 11.9 kg. In yearlings and adults, the proportion of females with two or more CL sharply increased with increasing body mass; however, when taking into account the effects of body mass (body mass is constant), there was a higher proportion of adults with two or more CL than yearlings. In adults, 54 does (5.4%) had more than two CL, showing strong body mass dependence: none of them had body mass <12.0 kg and 11% of does with eviscerated body mass >18.0 kg had three or more CL (S2 Table).

The synergistic effects of body mass and age on reproductive parameters were indicated by the homogeneity analysis (differences in the effects of body mass on fertility and potential litter size between yearlings and adults; Table 1) and confirmed by the GLM. In the GLMs, besides the main age category (yearlings *vs*. adults) and the effects of body mass *per se*, the interaction of body mass × age category also affected both fertility and potential litter size (Table 2). The effects of body mass on both reproductive parameters were more pronounced in yearlings than in adult does, and became weaker with age. The best GLM in adults predicts that potential litter size increases with body mass, but the effect is weaker in the first age category (young to middle-aged does) than in older adults (Table 3) where the potential litter size/body mass function is nearly flat (see also Fig 3).

Age-specific effects of body mass on potential reproductive output are clearly demonstrated in Fig 3. With increasing body mass from the lowest body mass class (average body mass of all females in this class: 11.9 kg) to the highest one (average body mass: 18.8 kg) the potential reproductive output in yearlings increased from 1.27 to 1.90 (by 50%). These differences were much smaller in adults: the number of CL in does from the lowest to the highest body mass class increased from 1.69 to 2.01 (by 19%) in young to middle-aged adults (estimated age: 2–7 years), and from 1.95 to 2.03 (only by 4%) in older adults. In young to middle-aged adults, the positive effect of body mass on potential reproductive output tended to cease at much lower values (approximately 15.5 kg) than in yearlings (17.0 kg), while in older individuals (estimated age: 8+ years) the number of CL did not differ much among body mass classes (Fig 3). This indicates that body mass influences primarily reproductive performance of younger, and in particular, lighter individuals.

## Discussion

In mammals, the onset of the first oestrous is generally strongly correlated with body mass or growth rate [12]. Thus, body mass would be expected to be the main factor determining the age of the first reproduction and in consequence also the fertility in yearlings; once past the threshold for puberty, body mass does not have any significant impact on fertility in adults (Table 1; Table 3). In populations of large herbivores, females generally produce first offspring at two or three years of age, but in some small- or medium-sized species (such as roe deer) females can give birth during or immediately after finishing their first year of life. Roe deer differ from other cervids by their high reproductive potential reflected in an early sexual maturity [48]. Mating of roe deer (the rut) occurs in mid to late summer [1] therefore the most common age at first breeding is 15–17 months. Indeed, in our study as many as 94 ± 1% of yearlings were fertile (Fig 1).

In our study, 50% of yearlings with eviscerated body mass <10.0 kg failed to ovulate while all yearlings heavier than 16.0 kg were fertile (S2 Table). Yearlings in poor body condition allocate their resources in body growth and development while heavier ones can allocate more in reproduction [23]. Due to lower body mass of yearlings in comparison with adults and their intensive growth in the 10-month period from mating to fawning, relative metabolic costs of the first reproduction are higher than in the subsequent reproductive years [22]. Except in the first part of pregnancy, mass-specific metabolism during gestation was higher in primiparous females than in multiparous individuals, indicating the occurrence of additional costs due to growth in young females (*ibid*.). Although we analysed reproductive capacity of roe deer females before implantation and ovulation *per se* is relatively low-cost process [32, 33], the average fertility rate of adult females was higher in comparison with yearlings at the same body mass, which may be the consequence of different metabolic costs between primiparous and multiparous females even at this stage of reproduction. That the trade-off between metabolic and reproductive costs drives fertility of roe deer females is confirmed also by the fact that no differences in fertility rates were found among adult does with presumably completed body growth neither after controlling for the body mass effects (Table 3).

The same age-dependent effect of body mass on fertility was observed in moose (*Alces alces*) in which ovulation rate was lower and more variable among primiparous females than in older cows, and at the same body mass prime-aged cows had higher probability to ovulate than yearlings [49, 50]. Connection between the onset of the first reproduction and body mass was reported also in some other ungulates, e.g. in red deer [51–59], fallow deer [60], reindeer (*Rangifer tarandus*) [61], white-tailed deer (*Odocoileus virginianus*) [62], wild boar (*Sus scrofa*) [63–65], and domestic sheep (*Ovis aries*) [66].

Body mass affects ovulation in roe deer females and even stronger the number of offspring that they can produce, which was clearly indicated also in our study. Our data on the effects of maternal body mass on both potential litter size and potential reproductive output fit well with existing findings, i.e. that young, primiparous females have on average smaller litters than adult does (e.g. [4]), and that roe deer females with higher body mass produce larger litters (e.g. [4, 6, 7, 12, 15]). [19] found in various populations from Great Britain that does carrying more than one CL were significantly heavier than does with a potential to produce singleton only; they also observed that the increase in potential litter size with body mass was particularly marked in yearlings which was confirmed also by our study (see Fig 3). In Scandinavia, roe deer females with above average body mass expressed 40% higher productivity (in number of fawns per doe) than those with a below average body mass [6]. Our results revealed that proportion of females carrying more than one CL markedly increased with the body mass in yearlings and in adult does (>90% of adults and all yearlings with eviscerated body mass >18.0 kg had potential to produce two or more offspring; S2 Table; Fig 1). However, at the same body mass yearlings produced fewer CL than adult does as also shown by [4].

Our data indicate that there is an age-dependent shift in reproductive potential and probably also in allocation of resources from body growth into reproduction. While young females (not only yearlings but also younger adults; see Fig 3) that have not reached their full body size allocate their resources primarily into the body growth and less into reproduction, older females exhibit larger reproductive efforts even when their body condition is weak. However, at higher body mass also younger individuals (including yearlings) shift their efforts into the reproduction, and after reaching an age-specific threshold the body mass does not have any further effects on the reproductive output of roe deer females (Fig 3). While in older adults body mass does not have any obvious influence on reproductive potential, in young adults this threshold seems to be at lower values as in yearlings. This indicates that subadult females balance reproductive efforts until reaching almost maximal body size, and that body mass primarily influences reproductive performance of younger, and in particular, lighter individuals. However, in young adult roe deer females body growth terminates at a specific age (24–26 months; [37, 48]) regardless of body size they reached, therefore even individuals of lower body mass, but with terminated body growth, may start allocating more in the reproduction.

By cross-sectional analysis we observed a marked decline of average body mass of roe deer females with aging, and elderly individuals on average weighed 1.7 kg (10.5%) less than middle-aged females (Fig 2). Although the observed decrease of average body mass with the age may partly be an artefact of potentially overestimated age in animals in poor condition due to their high tooth-wear rates (for the influence of individual and environmental factors on tooth-wear in different deer species, see review in [67]), many studies of wild mammals have also documented age-related changes in adult body mass which may decline through senescence of physiological function and reduced foraging ability (reviewed in [21]).

Considering the pronounced impact of body mass on reproductive potential of roe deer as well as a high rate of reproduction in early life it may be expected that reproductive potential of roe deer females would decrease toward the end of their life-span. However, our data showed only a slightly decreasing trend of potential reproductive output of very old females which was observed several years after body mass culmination (Fig 2). Actual litter sizes may partially differ from potential ones due to implantation failure and/or resorption of foetuses, especially in the oldest animals (see [19, 48]). Therefore, the existence of reproductive senescence in roe deer cannot be neither confirmed nor rejected based on our data.

So far, a study from France (Chizé) showed a marked decrease in fertility among roe deer females, older than 12 years [37]. Decrease in reproductive outcome with the senescence was more often observed in red deer (see [28]), and some studies suggested that hinds undergo reproductive senescence when they are 8–11 years old (Belgium: [35]), older than 12 years (Norway: [34, 68]) or older than 13 years (Slovenia: [64]). However, there was no observed senescence effect on reproduction of red deer hinds in Polish populations [58]. Similarly, there were no signs of senescence effect on reproduction of white-tailed deer females up to 15.5 years [62].

We analysed reproductive capacity of roe deer females in the early stage of pregnancy by counting CL. Since the ovulation is a relatively low-cost process [32, 33], CL counts are not convenient as a measure of reproductive effort/investment. Nevertheless, they still provide important insight in reproductive potential of roe deer females at an early stage of reproduction which predetermines also the reproductive effort in later, more advanced and costly stages. Indeed, our results fit fairly well with results of studies examining body mass effects in later stages of reproduction, i.e. by analysing foetuses or new-born fawns [4, 6, 12]. However, for determination of the age-related body mass effects on the reproductive performance, CL counts might even have an important pro: as they are measured in the early stage of the reproduction when the investment is rather low, female body mass is not yet affected by different investment (maternal care). Therefore, the influence of body mass on reproductive potential is likely clearer as in more advanced stages of reproduction.

Apart from body mass and age, population density and some environmental factors may also affect reproductive performance of roe deer females (for a review, see [3]). For example, pronounced inter-annual variability in reproductive performance of roe deer females (particularly yearlings) have already been observed in Slovenia [69]. Nevertheless, variable “year” was excluded from the majority of the best GLMs or its impact was weak in present study, indicating that this variable affected reproduction of roe deer females indirectly, through affecting their body mass. As such, it did not corrupt our results on the effects of body mass on reproductive potential of roe deer, but it still indicates that the inter-annual variability in environmental factors, i.e. in weather conditions or in availability of mast, and its indirect effects on reproductive performance has to be considered in the future. Moreover, up-following analyses should attempt to include also the influence of the most important population factors (e.g. density, health status, exposure to stress-inducing factors) and other environmental factors (e.g. environmental quality, interspecific interactions) to better understand the reproductive performance/success of roe deer females across the whole distribution range of the species. This information would be essential for understanding and predicting population dynamics in a wider spatiotemporal context, enabling more efficient management of roe deer populations with a diverse demographic structure and different reproductive performance.

## Supporting information

S1 Dataset Individual data on roe deer females (assessed age, body mass, number of corpora lutea in ovaries) used in the study. (XLSX)

S1 Text Corrections of body mass by removing the temporal effect. (DOCX)

S1 Table General Regression Model of body mass of roe deer females in Slovenia. The explanatory variables are year, age class, day in a year (covariate) and interaction year × age class; model was built using the best subset approach and Mallow’s Cp selection criterion; last level of each categorical variable served as contrast (estimate = 0) for the remaining levels of that variable; study period 2013–2015, n = 1312.
(DOCX)

S2 Table Percentage of females with given number of corpora lutea in different body mass classes. (DOCX)
