# Supplementary material for: Age-related effects of body mass on fertility and litter size in roe deer
Source: PLoS One. 2017 Apr 12;12(4):e0175579. doi: 10.1371/journal.pone.0175579 (PMC5389817; doi:10.1371/journal.pone.0175579)
Supplement: S1 Text — (DOCX) [file pone.0175579.s002.docx]

**S1 Text. Corrections of body mass by removing the temporal effect.**

Each year, samples were collected in the period of 4 months, from 1^st^ September to 31^st^ December. For each roe deer we recorded eviscerated carcass mass (total body mass less viscera but with head and feet on, measured with 0.5kg accuracy). Body mass of each animal was affected by animal’s age, year of sampling and individual characteristics. Besides that, measured body mass of each individual was also influenced by calendar date – the time in the year, when the individual was culled, which could disrupt the results of any further analyses. Therefore, we corrected all measurements of body mass by removing these temporal effects. We coded sampling (culling) date of each individual as a successive day in a year (where 1^st^ September was 244^th^ day, and 31^st^ December was 365^th^ day). We ran General Regression Model (GRM) with body mass as a dependent variable, and age class (yearlings, 2-year-olds, 3–4-year-olds, 5–7-year-olds, 8–9-year-olds, 10-year-olds and older), day in the year (covariate), interaction between day in the year × age class, year (categorical variable) and interaction year × age class as explanatory variables. To build all possible models, including all combinations of main effects and listed interactions, we used the best subsets approach. The best model was selected by the Mallow’s Cp criterion and is presented in Table 1. Based on this model, we estimated the effect of day in the year on individual body mass, using the formula (Δbody mass = β_day in the year_ (day in the year – mean day in the year)), where β is the parameter estimate for the effects of covariate “day of the year. We removed (substracted) the calculated temporal effect from the original body mass value. Using the described protocol, we standardised all body masses like if they were measured on the same (mean) sampled day in a year (this is the day 296, i.e. 23^rd^ October). All subsequent analyses were performed with the adjusted body mass values.
