# Supplementary material for: Age-related effects of body mass on fertility and litter size in roe deer
Source: PLoS One. 2017 Apr 12;12(4):e0175579. doi: 10.1371/journal.pone.0175579 (PMC5389817; doi:10.1371/journal.pone.0175579)
Supplement: S1 Table — The explanatory variables are year, age class, day in a year (covariate) and interaction year × age class; model was built using the best subset approach and Mallow’s Cp selection criterion; last level of each categorical variable served as contrast (estimate = 0) for the remaining levels of that variable; study period 2013–2015, n = 1312. (DOCX) [file pone.0175579.s003.docx]

**S1 Table.** **General Regression Model of body mass of roe deer females in Slovenia.** The explanatory variables are year, age class, day in a year (covariate) and interaction year × age class; model was built using best subset approach and Mallow’s Cp selection criterion; last level of each categorical variable served as contrast (estimate = 0) for the remaining levels of that variable; study period 2013–2015, n=1312.

| Variable | Level of variable | Estimate | Std. Err | p-value |
| --- | --- | --- | --- | --- |
| Year | 2013 | -0.177 | 0.115 | 0.125 |
|  | 2014 | 0.036 | 0.099 | 0.716 |
|  | *vs.* 2015 |  |  |  |
| Age class | 1 | -1.209 | 0.121 | <0.001 |
|  | 2 | 0.236 | 0.142 | 0.097 |
|  | 3–4 | 0.720 | 0.114 | <0.001 |
|  | 5–7 | 0.760 | 0.125 | <0.001 |
|  | 8–9 | 0.215 | 0.164 | 0.190 |
|  | *vs.* >10 |  |  |  |
| Day in a year | (covariate) | 0.015 | 0.001 | <0.001 |
| Year × age class | year 2013 & age class 1 | -0.310 | 0.185 | 0.095 |
|  | year 2013 & age class 2 | -0.099 | 0.217 | 0.647 |
|  | year 2013 & age class 3–4 | -0.141 | 0.177 | 0.425 |
|  | year 2013 & age class 5–7 | -0.091 | 0.187 | 0.628 |
|  | year 2013 & age class 8–9 | 0.412 | 0.271 | 0.129 |
|  | year 2014 & age class 1 | 0.204 | 0.162 | 0.208 |
|  | year 2014 & age class 2 | 0.328 | 0.196 | 0.094 |
|  | year 2014 & age class 3–4 | 0.249 | 0.153 | 0.103 |
|  | year 2014 & age class 5–7 | 0.005 | 0.170 | 0.978 |
|  | year 2014 & age class 8–9 | -0.502 | 0.206 | 0.015 |
|  | *vs.* year 2015 & age class >10 |  |  |  |
| Intercept |  | 10.804 | 0.430 | <0.001 |
