# Supplementary material for: Age-related effects of body mass on fertility and litter size in roe deer
Source: PLoS One. 2017 Apr 12;12(4):e0175579. doi: 10.1371/journal.pone.0175579 (PMC5389817; doi:10.1371/journal.pone.0175579)
Supplement: S2 Table — (DOCX) [file pone.0175579.s004.docx]

**S2 Table. Percentage of females with given number of corpora lutea in different body mass classes.**

|  | **Yearlings (%)** | | | | | **Adults (%)** | | | | | |
| --- | --- | --- | --- | --- | --- | --- | --- | --- | --- | --- | --- |
|  | **Number of corpora lutea** | | | | | **Number of corpora lutea** | | | | | |
| **Body mass (kg)** | **0** | **1** | **2** | **3** | **N** | **0** | **1** | **2** | **3 or**  **more** | **N** |  |
| **<10.0** | 50.0 | 38.9 | 11.1 | 0 | 18 | 0 | 42.9 | 57.1 | 0 | 7 |  |
| **10.0–11.9** | 6.8 | 59.1 | 31.8 | 2.3 | 44 | 8.1 | 24.3 | 67.6 | 0 | 37 |  |
| **12.0–13.9** | 3.2 | 51.1 | 45.7 | 0 | 94 | 1.2 | 21.3 | 74.0 | 3.6 | 169 |  |
| **14.0–15.9** | 3.1 | 34.0 | 62.9 | 0 | 97 | 1.0 | 15.8 | 79.2 | 4.0 | 298 |  |
| **16.0–17.9** | 0 | 17.4 | 82.6 | 0 | 46 | 0.3 | 10.2 | 84.6 | 4.9 | 305 |  |
| **>18.0** | 0 | 0 | 100.0 | 0 | 6 | 2.6 | 3.7 | 82.7 | 11.0 | 191 |  |
| **All together** | **5.9** | **40.0** | **53.8** | **0.3** | **305** | **1.4** | **13.2** | **80.0** | **5.4** | **1007** |  |
